# Examination of Ataxin-3 (atx-3) Aggregation by Structural Mass Spectrometry Techniques: A Rationale for Expedited Aggregation upon Polyglutamine (polyQ) Expansion* [Fn FN2]

**DOI:** 10.1074/mcp.M114.044610

**Published:** 2015-02-20

**Authors:** Charlotte A. Scarff, Bruno Almeida, Joana Fraga, Sandra Macedo-Ribeiro, Sheena E. Radford, Alison E. Ashcroft

**Affiliations:** From the ‡Astbury Centre for Structural Molecular Biology, School of Molecular and Cellular Biology, University of Leeds, Leeds, LS2 9JT, UK;; §IBMC-Instituto de Biologia Molecular e Celular, Universidade do Porto, 4150–4180 Porto, Portugal

## Abstract

Expansion of polyglutamine stretches leads to the formation of polyglutamine-containing neuronal aggregates and neuronal death in nine diseases for which there currently are no treatments or cures. This is largely due to a lack in understanding of the mechanisms by which expanded polyglutamine regions contribute to aggregation and disease. To complicate matters further, several of the polyglutamine-disease related proteins, including ataxin-3, have a multistage aggregation mechanism in which flanking domain self-assembly precedes polyglutamine aggregation yet is influenced by polyglutamine expansion. How polyglutamine expansion influences flanking domain aggregation is poorly understood. Here, we use a combination of mass spectrometry and biophysical approaches to investigate this issue for ataxin-3. We show that the conformational dynamics of the flanking Josephin domain in ataxin-3 with an expanded polyglutamine tract are altered in comparison to those exhibited by its nonexpanded counterpart, specifically within the aggregation-prone region of the Josephin domain (amino acid residues 73–96). Expansion thus exposes this region more frequently in ataxin-3 containing an expanded polyglutamine tract, providing a molecular explanation of why aggregation is accelerated upon polyglutamine expansion. Here, harnessing the power of ion mobility spectrometry-mass spectrometry, oligomeric species formed during aggregation are characterized and a model for oligomer growth proposed. The results suggest that a conformational change occurs at the dimer level that initiates self-assembly. New insights into ataxin-3 fibril architecture are also described, revealing the region of the Josephin domain involved in protofibril formation and demonstrating that polyglutamine aggregation proceeds as a distinct second step after protofibril formation without requiring structural rearrangement of the protofibril core. Overall, the results enable the effect of polyglutamine expansion on every stage of ataxin-3 self-assembly, from monomer through to fibril, to be described and a rationale for expedited aggregation upon polyglutamine expansion to be provided.

Polyglutamine (polyQ)^1^ diseases comprise a group of hereditary neurodegenerative disorders in which expansion of polyQ stretches within their causative proteins induces protein aggregation and the formation of polyQ-containing neuronal aggregates (1). The mechanisms by which expanded polyQ regions contribute to aggregation and disease are not well understood. In all cases, polyQ length is negatively correlated with the age of onset of the disease (2), but the various polyQ disorders are associated with different neurodegenerative symptoms and affect different regions of the brain (3). Several of the polyQ proteins, including ataxin-3 (atx-3) (4) and huntingtin (5), have been shown to aggregate *in vitro* through a complex multidomain misfolding pathway (6) in which flanking domain aggregation precedes polyQ aggregation. Increasing evidence also suggests a key role for misfolding of flanking regions in the process of polyQ aggregation *in vivo* (7–10). Thus, as the proteins have no sequence similarity other than in their polyQ regions, flanking domain content may be significant in determining the disease state and neuronal-specific selectivity. Given that there is growing support to suggest that the toxic entities in polyQ diseases are the soluble oligomers and assembly intermediates, rather than the fibrillar aggregates (11), effective therapeutics may be generated by targeting flanking domain interactions (12) rather than targeting the polyQ region itself. An enhanced understanding of the molecular mechanisms of assembly of polyQ proteins is required, as is a greater comprehension of the effects of polyQ length on the structure, dynamics, aggregation propensity, and oligomerisation pathway of the flanking domains. Here, we set out to determine the influence of an expanded polyQ tract on each stage of atx-3 aggregation by harnessing the power of mass-spectrometry-based approaches to identify and characterize assembly mechanisms (13, 14).

Atx-3 consists of a structured N-terminal Josephin domain (JD), which has ubiquitin protease activity (15) and an intrinsically disordered C-terminal region, the latter containing several ubiquitin-interacting motifs (UIMs) followed by the polyQ tract and a variable region (16) (Fig. 2*A*). *In vivo*, expansion of the polyQ stretch beyond ca. 55 glutamine residues results in Machado–Joseph disease (17). Consistent with this, atx-3 with a polyQ tract beyond ca. 55 glutamine residues aggregates into amyloid-like fibrils rapidly *in vitro* (18). Aggregation proceeds by means of a two-stage pathway (4): the first stage resulting in the production of SDS-sensitive, short, curvilinear, protofibrils, and the second producing long-straight and SDS-resistant mature fibrils. The first stage involves self-association of the JD (19) and occurs in all atx-3 variants whether or not they contain a polyQ region of nonpathological length (nonexpanded, 12–40 glutamine residues (17)), an expanded polyQ region of disease length (polyQ-expanded, 55–84 glutamine residues (17)), or are devoid of a polyQ region (20). The second stage occurs only in polyQ-expanded atx-3 and involves hydrogen bonding between side-chains in the polyQ region (21), which renders aggregation irreversible.

Despite the fact that the first stage of atx-3 aggregation does not require the polyQ tract, aggregation of polyQ-expanded atx-3 occurs more rapidly (with a shorter lag time) than aggregation of nonexpanded atx-3 (4, 20). The precise molecular mechanism for this observation has yet to be elucidated. An initial hypothesis was that polyQ expansion destabilizes atx-3, allowing the JD to adopt misfolded, aggregation-prone conformations more readily (15, 18). However, a study comparing atx-3 constructs with polyQ regions of different lengths showed that polyQ expansion does not affect the folding/unfolding kinetics or thermodynamic stability of the JD (22). Consequently, it has been postulated that the expanded polyQ tract may perturb the structure of the JD without affecting its stability (20).

We set out to address why aggregation occurs more rapidly in atx-3 with an expanded polyQ tract by studying monomeric, oligomeric, and fibril structures for atx-3 with a pathological length polyQ tract of 77 glutamines with a single, naturally occurring, lysine residue in the fourth position (named atx-3(78Q)); atx-3 with a nonpathological length polyQ tract of 13 glutamines (also with a single lysine residue in the fourth position) (atx-3(14Q)); and the isolated JD. Results from a combination of electrospray ionization-ion mobility spectrometry-mass spectrometry (ESI-IMS-MS), limited proteolysis, fluorescence spectroscopy, and transmission electron microscopy (TEM) analyses confirm that protofibrils of these three atx-3 constructs are formed through equivalent processes and reveal that the resulting protofibril cores are similar, if not identical. Limited proteolysis experiments combined with MS analyses provide evidence that an expanded polyQ tract alters the conformational dynamics of the JD, exposing its aggregation-prone region more frequently than in its nonexpanded counterparts, rationalizing the enhanced aggregation potential of the polyQ-expanded protein. Finally, oligomers populated *en route* to fibrils are examined by ESI-IMS-MS and a model for oligomer growth is provided. Together these results reveal how polyQ length affects each stage of atx-3 aggregation and demonstrate how different MS-based techniques can provide information about each stage of the aggregation mechanism.

## EXPERIMENTAL PROCEDURES

### 

#### 

##### Protein Preparation

cDNAs encoding human ataxin-3 (14Q) (isoform 2 ((16) (P54252–2)), the JD (atx-3 residues 1–182), and polyQ-expanded atx-3 (78Q) (isoform 2, G→R directly following polyQ tract (VAR 013689) (23)) were subcloned into pDEST17 plasmid vectors (19). Upstream of the coding site, a sequence coding for an N-terminal hexa-histidine tag and a linker region containing a cleavage site for the recombinant tobacco etch virus protease were incorporated. The proteins were expressed in *Escherichia coli* strains BL21(DE3)-pLysS or BL21-SI and soluble protein purified by nickel-affinity chromatography followed by gel-filtration chromatography. Purified protein samples were snap-frozen and stored at −80 °C. Each recombinant protein construct retains the hexa-histidine tag and linker region (SYYHHHHHHLENLYFQG) as removal of this from the atx-3(78Q) construct led to insolubility. The presence of the hexa-histidine tag and linker region did not significantly affect the CCSs measured for the JD or atx-3(14Q) by IMS-MS nor the observed products of limited proteolysis. Protofibril structures formed were also not effected (data not shown). Protein samples were buffer exchanged into 250 mm ammonium bicarbonate, 1 mm DTT, pH 8.1, for all aggregation studies by use of ZEBA^TM^ desalting columns (Thermo Fisher Scientific, Waltham, MA, USA), prepared at a concentration of 20–40 μm and used immediately. A slightly basic pH was used to ensure that the pH remained constant during the time-course of aggregation using the volatile buffers required for ESI-MS. Under these conditions, atx-3 aggregates by the same two-step mechanism observed in nonvolatile buffers at pH 7.4 (20).

##### ESI-(IMS)-MS Analysis

A Synapt High Definition Mass Spectrometer (HDMS) quadrupole time-of-flight mass spectrometer (Waters Corporation, Manchester, UK) was used to analyze all samples. Samples were introduced into the instrument by direct infusion nanoESI with in-house prepared gold-coated borosilicate glass capillaries. MS and IMS-MS spectra were recorded in nanoESI positive mode using the following instrument parameters: cone voltage 30–60 V; source temperature 60 °C; backing pressure 3.5–4.5 mBar; traveling wave height 8 V; traveling wave speed 300 m/s; IMS gas flow 20 ml/min. Data were processed by use of MassLynx v4.1 and Driftscope software supplied with the mass spectrometer. Driftscope plots show drift time on the *x* axis, *m/z* on the *y* axis, and relative ion intensity on the *z* axis (square-root scale). Estimated CCSs for different species were calculated by use of a calibration curve using beta-lactoglobulin, avidin, alcohol dehydrogenase, and concanavalin A, following the protocol previously described (24).

##### Fibril Growth Experiments

Thioflavin T (ThT) fluorescence was recorded on a FLUOstar Omega reader (BMG, Labtech Gmbh, Ortenberg, Germany) from a 96-well black-wall plate (Costar^TM^, Tewkesbury, MA, USA) sealed with clear sealing film. Samples of 100 μl volume contained 40 μm protein in 250 mm ammonium bicarbonate, 1 mm DTT, 20 μm ThT, pH 8.1, and were incubated at 37 °C without shaking. Fluorescence measurements were acquired for three or more replicates of each sample over 48 h. Samples were incubated for a further 48 h at 37 °C. Samples from the growth assays were examined on freshly ionized carbon- and formvar-coated TEM grids negatively stained with 2% (w/v) uranyl acetate by use of a JEM-1400 microscope (JEOL, Tokyo, Japan). Aliquots of samples were removed at various time points through aggregation for analysis by ESI-IMS-MS in real time.

##### Analysis of JD Peptides and Cross-Seeding Experiments

Peptides composed of JD residues 48–59, 73–96, 144–154, and 159–167 (with N-terminal acetylation and C-terminal amidation) were purchased from Biosynthesis Inc. (Lewisville, TX) Peptides were incubated at 20 μm concentration in 250 mm ammonium bicarbonate, 1 mm DTT, 20 μm ThT, pH 8.1, for 24 h at 37 °C without shaking after which time ThT fluorescence was measured on a plate reader and TEM performed as above. The ThT fluorescence of the peptides was also monitored, immediately after their dilution into buffer, over 24 h under the conditions stated above. For cross-seeding experiments, assays were performed as described above but using 20 μm protein in the presence of a 1:1 molar ratio of JD peptide. Boxplots were created by use of the web tool BoxPlotR (25).

##### Limited Proteolysis-Fibrils

For limited proteolysis experiments on fibrils, fibrillar material formed was collected by centrifugation (16,000 *g*, 30 min) and resuspended in fresh buffer as described above. Proteinases, bovine trypsin, or proteinase K (Sigma Aldrich), were added at 1:100 or 1:50 proteinase to protein molar ratios, respectively, and proteolysis was allowed to proceed for 30 min at 37 °C before reactions were quenched by the addition of 10% (v/v) formic acid. Fibrillar material remaining after digestion was separated from soluble material, again by centrifugation (16,000 *g*, 30 min). The insoluble fraction was depolymerized in 100% (v/v) hexafluoroisopropanol (for 3 days at 300 rpm, 37 °C). Fibrillar samples were air dried and redissolved in 50:48.8:0.2 acetonitrile/water/formic acid (v/v/v) prior to ESI-MS analysis. Supernatant samples were analyzed directly by ESI-MS.

##### Limited Proteolysis-Monomers

For limited proteolysis experiments on monomeric samples (20 μm concentration), bovine trypsin was added at 1:100 proteinase:protein molar ratio and samples incubated for 15 min at 37 °C prior to ESI-MS analysis. To monitor limited proteolysis over time for the JD, atx-3(14Q), and atx-3(78Q), bovine trypsin was added at 1:10 (w/w) proteinase to protein ratio and samples incubated for 1 min, 4 min, and 8 min at 37 °C prior to ESI-MS analysis in real time. A peptide standard (MAEGGVTSEDYR, N-terminally acetylated and C-terminally amidated) was added to each protein construct prior to the addition of trypsin in a 5:1 protein:peptide molar ratio. The relative intensities of tryptic peptide ions were normalized to the intensity of the peptide standard in each case to allow for relative quantification of the rates of tryptic peptide production. Tryptic peptides resulting from limited proteolysis of the JD and atx-3 were separated by IMS and further characterized by collision-induced dissociation (CID) MS/MS sequencing when required. Experiments were conducted in triplicate and representative spectra are shown.

##### Modeling

Isotropic assembly (cross-section Ω is related to the oligomeric state n by Ω_monomer_ × n^2/3^) and linear assembly models of oligomers (Ω = an + k, where a = area added per monomer subunit and k is related to the fibril cap) were produced as described previously (14). The ring structures were calculated following the method used by Bernstein *et al.* (26). The MOBCAL algorithm was modified to allow for coarse-grain modeling of a complex as described previously (27). The CCS measurements obtained for the most compact JD oligomers observed were used in the modeling calculations (lowest charge state observed for each oligomer). Alternatively, the values for the most highly populated oligomer charge states did not affect the model to which the data best fitted.

## RESULTS

### 

#### 

##### An Expanded PolyQ Tract Does Not Alter Protofibril Structure

Although the second stage of aggregation has been attributed to intermolecular hydrogen bonding in the expanded polyQ tract (21), the molecular architectures of fibrils formed from atx-3 have not been reported in any detail to date. To improve our understanding, we used limited proteolysis combined with ESI-MS to elucidate the regions of atx-3 involved in the protected core of JD, atx-3(14Q), and atx-3(78Q) protofibrils, as well as atx-3(78Q) mature fibrils.

**Fig. 1.**
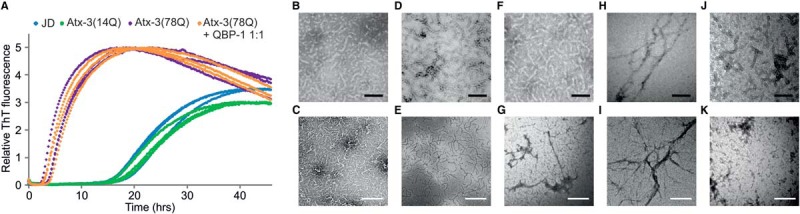
**Fibril formation of atx-3.** (*A*) Fibril growth of atx-3 constructs, the JD, atx-3(14Q) and atx-3(78Q), monitored by ThT fluorescence (40 μm protein, pH 8.1, 37 °C, three representative traces of each sample are shown). TEM images of (*B*, *C*) the JD, (*D, E*) atx-3(14Q), and (*F, G*) atx-3(78Q) samples shown in a recorded after 48 h incubation and TEM images of (*H, I*) atx-3(78Q) and (*J, K*) atx-3(78Q) + QBP-1 1:1 molar ratio recorded after 96 h incubation. Images are shown at two different magnifications (black scale bars 100 nm, white scale bars 500 nm).

The aggregation reactions of the JD, atx-3(14Q), and atx-3(78Q) were monitored by ThT fluorescence and TEM over time. ThT fluorescence increases upon binding to beta-rich structures and reports on the first stage of atx-3 aggregation. The second polyQ-dependent step of aggregation does not affect ThT fluorescence and proceeds after the initial ThT increase is observed (4). Consistent with previous observations (4, 20), ThT fluorescence kinetics indicated that protofibril formation occurs more rapidly for atx-3(78Q) than for atx-3(14Q) or the JD (Fig. 1*A*). The appearance of short, curvilinear protofibrils, with similar morphologies was observed for all three constructs by TEM after 48 h incubation (Figs. 1*B*-1*G*). A small proportion of long-straight fibrils was observed in the atx-3(78Q) sample at this time point (Fig. 1*G*). Full maturation of atx-3(78Q) protofibrils into SDS-resistant fibrils was observed after 96 h incubation (Figs. 1*H* and 1*I*). Mature fibrils were SDS-insoluble (measured by SDS-PAGE (not shown)) and TEM analysis showed no evidence for the presence of protofibrils in this fibril sample. Glutamine Binding Protein (QBP-1) (28), an 11-residue peptide that inhibits the second stage of aggregation of polyQ-expanded atx-3 while not affecting protofibril formation (4), was used to stall the aggregation of atx-3(78Q) at the protofibril stage. Atx-3(78Q) incubated in a 1:1 molar ratio with QBP-1 was observed to undergo very similar aggregation kinetics to those of atx-3(78Q) alone (Fig. 1*A*) and formed protofibrils that did not mature into SDS-insoluble fibrils after 96 h incubation (Figs. 1*J* and 1*K*).

**Fig. 2.**
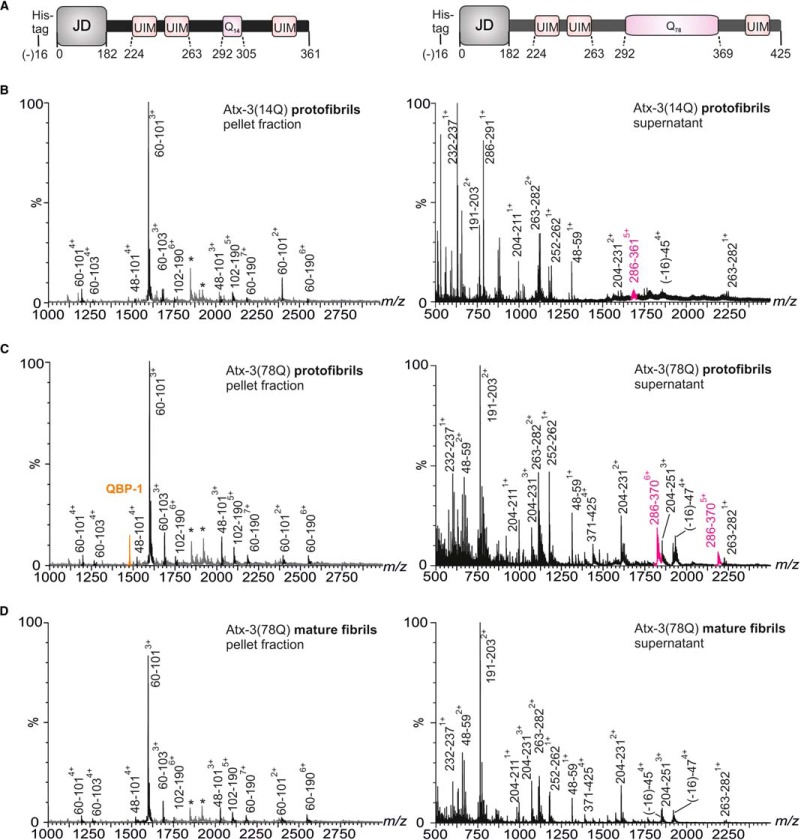
**Limited proteolysis of protofibrils and mature fibrils.** (*A*) Schematic illustrations of atx-3(14Q) (left) and atx-3(78Q) (right) with amino acid residue numbers for each domain shown. Mass spectra obtained following limited proteolysis with trypsin of (*B*) atx-3(14Q) protofibrils (*C*) atx-3(78Q) protofibrils and (*D*) atx-3(78Q) mature fibrils. Mass spectra of (left) the depolymerized fibrillar material are contrasted with those obtained from analysis of (right) the soluble products of proteolysis. Asterisks represent species observed in the pellet fraction that were also observed in supernatant samples ((-16)-45^4+^ and (-16)-47^4+^, respectively). Peaks identified as containing the polyQ tract are highlighted in pink, while those representing QBP-1 are highlighted in orange.

To determine the regions of atx-3 involved in the protofibril and mature fibril cores, limited proteolysis with trypsin was performed on the fibrillar material obtained from each construct. Proteolysed fibrillar material was separated from the soluble products of proteolysis by centrifugation, and the pellet and supernatant fractions were analyzed by ESI-MS. The pelleted fraction was depolymerized in 100% hexafluoroisopropanol prior to MS analysis (see Methods). Mass spectra obtained from analysis of the depolymerized fibrillar material for atx-3(14Q) and atx-3(78Q) protofibrils trapped by the addition of QBP-1 were indistinguishable, indicating that the regions of atx-3 involved in protofibril formation are the same, irrespective of the length of the polyQ tract (Figs. 2*A*-2*C*). The peptide species identified consisted of residues 48–101, 60–101, 60–103, 102–190, and 60–190 (the JD consists of residues 1–182). Peptides spanning residues 60–190 were identified in the mass spectra of the depolymerized fibrillar material but were not found in the spectra of the soluble products of proteolysis. This suggests that the protected core of the protofibrils lies between residues 60 and 190. The polyQ regions of atx-3(14Q) and atx-3(78Q) were cleaved from the protofibril core upon limited proteolysis of their respective protofibrils and were observed in mass spectra of the soluble supernatant fraction, illustrating, as elucidated previously (4), that the polyQ stretch is not structurally involved in the protofibril core (Figs. 2*B* and 2*C*). Interestingly, the mass spectrum obtained of the atx-3(78Q) mature fibril pellet fraction (Fig. 2*D*) was similar to that obtained for the atx-3(78Q) protofibril core (Fig. 2*C*). SDS-PAGE analysis revealed that a significant amount of SDS-insoluble material remained after hexafluoroisopropanol treatment of the mature atx-3(78Q) fibrils, presumably consisting of SDS-resistant polyQ peptide sequences (data not shown) that were not depolymerized by hexafluoroisopropanol. Mass spectra obtained of the soluble products of proteolysis from atx-3(78Q) protofibrils and mature fibrils were also remarkably similar (Figs. 2*C* and 2*D*), but fragments containing residues 286–370 were not observed in mass spectra obtained following limited proteolysis of the mature fibrils. This suggests that this region, which consists of six residues N-terminal of the polyQ tract, the expanded polyQ tract, and a C-terminal arginine residue, forms SDS-insoluble aggregates. Together, these data indicate that the mature fibrils consist of two distinct structured regions, the protofibril core and a polyQ-containing core, that can be separated by proteolysis. Moreover, the results imply that involvement of the polyQ stretch in formation of SDS-resistant, mature fibrils occurs as a distinct second step in which structural rearrangement of the protofibril core is not required.

Limited proteolysis of the protofibrils was performed additionally with proteinase K, a nonspecific protease, and depolymerized protofibrillar material was analyzed by MS. Again, the major peptides identified within the depolymerized protofibrillar fractions consisted of residues between 60 and 190 (more specifically, between residues 62 and 183) (supplemental Fig. 1). This supports the conclusion that the cores of the JD, atx-3(14Q), and atx-3(78Q) protofibrils are similar, if not identical.

##### Global Monomeric Conformation Is Not Significantly Affected by an Expanded PolyQ Tract

**Fig. 3.**
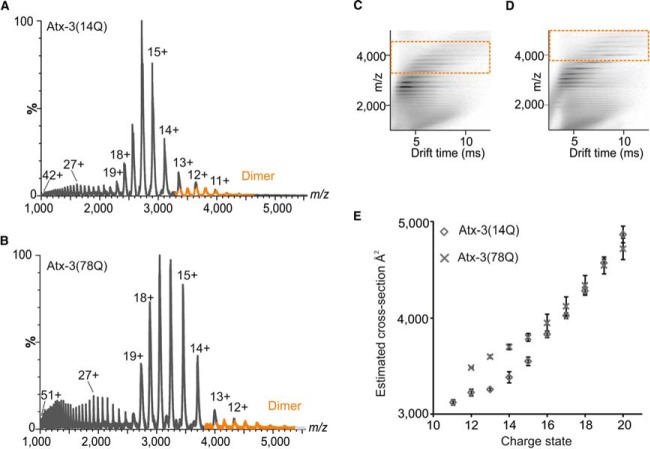
**Monomeric conformations of atx-3.** Representative ESI mass spectra of (*A*) atx-3(14Q) and (*B*) atx-3(78Q) obtained at t = 0 with (*C, D*) respective driftscope plots. Monomeric ion species are shown in black, and dimeric ion species are highlighted in orange. (*E*) Estimated CCSs obtained for monomeric ion species (compact charge state distribution) of atx-3(14Q) (224) and atx-3(78Q) (×). Average measurements from three datasets are shown with error bars representing the S.E. in each case.

In previous work, using ESI-IMS-MS and limited proteolysis (29), we showed that atx-3(14Q) is globally more dynamic than the JD, exhibiting a broader array of conformers, attributable to dynamic movement and flexibility within the C-terminal polyQ-containing region of atx-3 (29). Here, we compared the conformational properties of atx-3(14Q) and atx-3(78Q) using ESI-IMS-MS (Fig. 3).

Under carefully controlled solvent and instrumental conditions, gas-phase measurements of protein conformations can be reflective of solution-phase structures (30, 31) and the extent of ionization during the ESI process is reflective of surface-exposed area and mass (32). ESI mass spectra of atx-3(14Q) (Fig. 3*A*) and atx-3(78Q) (Fig. 3*B*) contain multiple charge state distributions (CSDs), one representing more compact ion species (charge states 11+ to 19+ and 12+ to 20+, respectively) and several representing more extended ion conformations (charge states 20+ to 42+ and 21+ to 51+, respectively). These spectra are typical for proteins that contain intrinsically disordered regions and that adopt a wide range of conformational states with different surface-exposed areas (33).

IMS can be used in conjunction with MS to study the relative size (rotationally averaged CCS) and distribution of conformations adopted by a protein species. Representative three-dimensional ESI-IMS-MS datasets of atx-3(14Q) and atx-3(78Q) indicate that these atx-3 constructs adopt a range of conformations of similar size, despite differing by 8 kDa in molecular mass (Figs. 3*C* and 3*D*, respectively). Ion mobility arrival time distributions (ATDs) for individual charge states of each protein species were extracted from the three-dimensional ESI-IMS-MS datasets, and modal drift times were used to estimate a CCS for each ion species (Fig. 3*E*). At the lowest charge states observed (11+ to 15+), atx-3(14Q) conformers had shorter drift times and smaller CCSs than the equivalent atx-3(78Q) conformers. These conformers are each ∼20% larger in size than expected if atx-3(14Q) and atx-3(78Q) exhibited compact globular structures (based on their mass and an average density of 0.44 Da/Å^3^) (34). The difference in CCS measurement for these two species is consistent with that expected for a species with an increase in mass of 8 kDa (*i.e.* an extra 64Q residues) exhibiting a globular/compact conformation. The wide spread of charge state ions with their associated CCS measurements exhibited by both atx-3 species, and the presence of multiple CSDs within their MS spectra with higher intensity at lower charge states supports the notion that the proteins have both structured and disordered regions (35). (For charge states 16 + to 20 +, the global conformations populated by atx-3(78Q) are as compact as those populated by atx-3(14Q). This indicates that there is less disorder in atx-3(78Q) than in atx-3(14Q) for these species and thus suggests that the expanded polyQ region is in a compact conformation that is more stable and less prone to unfolding than other parts of the atx-3 molecule.)

##### The Conformational Dynamics of the JD Are Altered in PolyQ-Expanded Atx-3

**Fig. 4.**
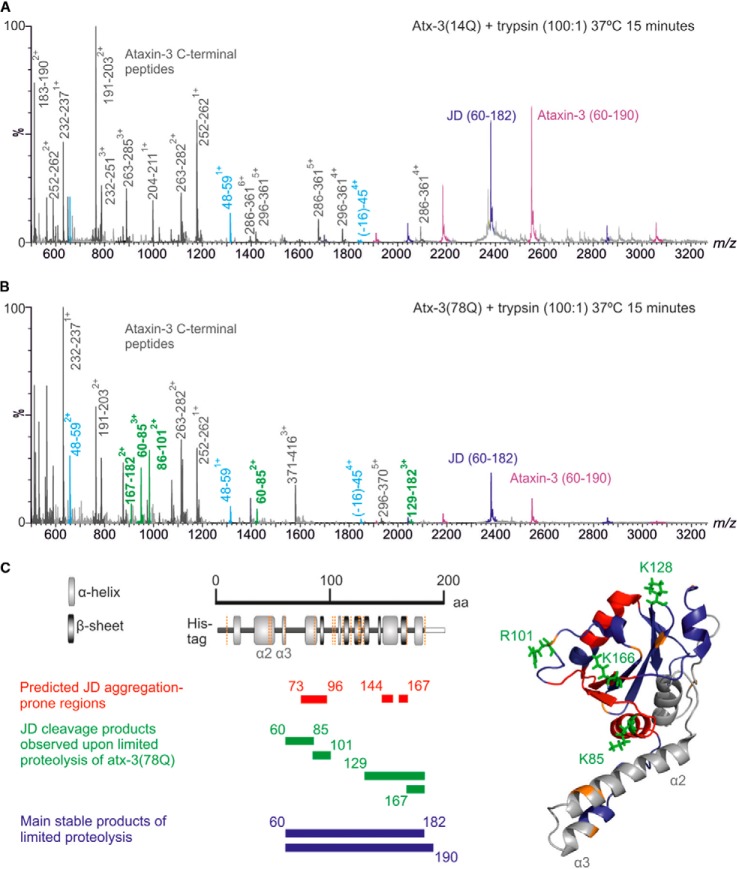
**Limited proteolysis of atx-3 monomers.** Representative ESI mass spectra of (*A*) atx-3(14Q) and (*B*) atx-3(78Q) obtained following limited trypsinolysis. Samples were exposed to trypsin for 15 min at 37 °C. Major stable products of proteolysis were JD residues 60–182 (dark purple) and atx-3 residues 60–190 (light purple). The C terminus of atx-3 was digested rapidly, producing a multitude of peptides (dark gray) and cleavage also occurred at sites within the dynamic α2/α3 hairpin of the JD (peptides in light blue). Additional fragments produced by cleavage at sites within the JD were observed for atx-3(78Q) (green). A list of all peptides identified is given in supplemental Table 1. (*C*) Schematic illustrating correlation between predicted aggregation-prone regions of the JD (34) (red), additional cleavage products observed for atx-3(78Q) (green) and the major stable products of proteolysis (purple). Amino acid numbers and secondary structure elements of the JD are shown with potential trypsin cleavage sites in orange. Key residues are identified on a ribbon diagram of the JD (PDB Number:1YZB (32)).

As the global conformations adopted by atx-3(14Q) and atx-3(78Q) in their monomeric states were observed to be similar by ESI-IMS-MS, we next investigated whether the local conformational dynamics of the JD are perturbed upon polyQ expansion. Atx-3(14Q) and atx-3(78Q) were each subjected to limited proteolysis with trypsin and the resulting products identified by ESI-IMS-MS in real time. The C-terminal intrinsically disordered region of atx-3 in both constructs was digested rapidly, and cleavage also occurred at sites within the dynamic α2/α3 hairpin of the JD (at residues R45, R47, and R59, leading to fragments (-16)-45 and 48–59) (Figs. 4*A* and 4*B*). The JD core region was not unfolded by these cleavage events and accumulated as a stable product of limited proteolysis (60–182) (also the stable product incorporating residues 60–190 was formed). The observation of the same major stable proteolysis products for both atx-3(14Q) and atx-3(78Q) suggests that each protein has similar conformational stability, consistent with previous results (22). Importantly, however, several peptides originating from the JD were observed when atx-3(78Q) was digested (60–85, 86–101, 129–182, 167–182) that were not observed for atx-3(14Q) (Figs. 4*B* and 4*C*). Accordingly, residues K85, R101, K128, and K166 were exposed (or became exposed) for a sufficiently long time period to allow trypsin cleavage in atx-3(78Q) but were not exposed for a sufficient time for cleavage to occur in atx-3(14Q) under the conditions studied.

The α2/α3 hairpin of the JD is the only highly dynamic region of the JD exhibiting extensive local motions (as judged by NMR relaxation measurements (36, 37)). K85 interacts with this region, and so this residue could become exposed transiently to proteinase during native dynamic movement of the JD (Fig. 4*C*). By contrast, residues R101, K128, and K166, while surface-exposed (Fig. 4*C*), are not within dynamic regions (36) and so must be involved in local interactions that restrict their cleavage. For JD cleavage products to be observed upon limited proteolysis of atx-3(78Q) that are not observed for atx-3(14Q), two scenarios can be proposed. The first is that the JD populates a unique native-like conformation in the presence of an expanded polyQ tract that is distinctively susceptible to limited proteolysis. The second is that the JD populates the same conformational ensemble in the presence of an expanded polyQ tract but that the rate of conformational transition between native-like states is altered such that the susceptibility to proteolysis of K85 is enhanced. In the latter case, the limited proteolysis data can be explained if cleavage occurs at K85 first, resulting in unfolding and exposure of residues R101, K128, and K166 for subsequent cleavage. Consistent with this proposal, peptides 86–101, 129–182, or 167–182 are not observed before the appearance of peptide 60–85.

**Fig. 5.**
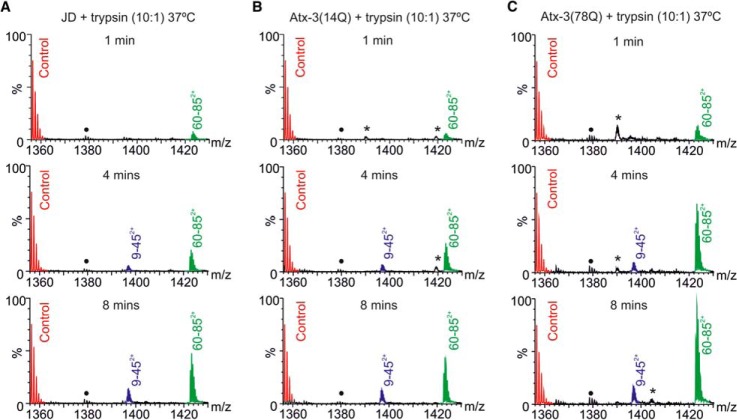
**Limited proteolysis of atx-3 constructs over time.** Representative ESI mass spectra of (*A*) JD, (*B*) atx-3(14Q), and (*C*) atx-3(78Q) obtained following exposure to trypsin (10:1 protein: enzyme molar ratio) at 37 °C for (top) 1 min, (middle) 4 min and (bottom) 8 min, respectively. Spectra are normalized to the relative intensity of a control peptide (JD 48–59) added at a 5:1 protein: peptide molar ratio. (*) Partially digested fragments of atx-3 (●) Sodiated peptide ions.

To distinguish between these scenarios, the appearance of the peptide 60–85 upon limited proteolysis of the JD, atx-3(14Q) and atx-3(78Q) was monitored in real time by use of ESI-MS. We posited that if atx-3(78Q) populates a unique conformation that exposes additional JD residues for cleavage, then K85 should not be cleaved in atx-3(14Q) or the JD. By contrast, if conformational dynamics between native-like states are altered in atx-3(78Q), then cleavage at K85 should occur in atx-3(14Q) and the JD but on a slower time scale. The results showed that the peptide 60–85 became populated upon exposure to trypsin in all three samples but appeared at a much faster rate for atx-3(78Q) than for atx-3(14Q) or the JD alone (Fig. 5). Conversely, the JD peptide residues 9–45 became populated at a similar rate for all three constructs. This suggests that the region of the JD surrounding K85 is more exposed, on average, in atx-3(78Q) than in atx-3(14Q) or the JD, reflecting an altered rate of conformational transition between native-like states for the expanded construct in comparison to the JD and atx-3(14Q). The extent of any conformational change resulting in <5% difference in CCS may affect changes in proteolysis but would not be detected by IMS-MS analyses.

**Fig. 6.**
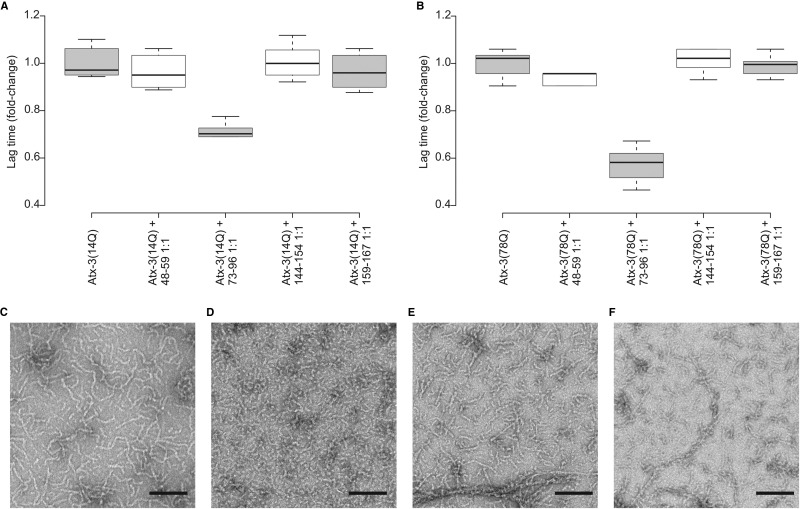
**Fibril formation of atx-3 in the presence of JD peptides.** The effect of the presence of the JD peptides 48–59, 73–96, 144–154 and 159–167 on the lag time of fibril formation for (*A*) atx-3(14Q) and (*B*) atx-3(78Q) (*n* = 6, 1:1 protein: peptide molar ratio, 20 μm protein in 250 mm ammonium bicarbonate, 1 mm DTT, pH 8.1, 37 °C without shaking) and corresponding TEM images taken after 48 h incubation for (*C*) atx-3(14Q), (*D*) atx-3(14Q) + 73–96 1:1, (*E*) atx-3(78Q) and (*F*) atx-3(78Q) + 73–96 1:1. Scale bars = 100 nm.

Amyloid fibril formation is thought to be driven by the exposure of short aggregation-prone regions (38), and the region 73–96, surrounding K85 in the JD, is one of three JD regions (73–96, 144–154, and 159–167) predicted to be aggregation prone by computational analysis (39). To determine whether enhanced exposure of residues 73–96 affects fibril formation, the aggregation behavior of this region was assessed as an isolated synthetic peptide, as well as its influence on atx-3 aggregation. We hypothesized that if exposure of the region 73–96 is necessary for fibril formation, then the aggregation kinetics of atx-3 should be altered in the presence of this peptide. Peptides composed of JD residues 73–96, 144–154, and 159–167 were synthesized and incubated under conditions identical to those used to study the aggregation of atx-3 (see Methods). These peptides all formed ThT-positive fibrils immediately upon dilution from DMSO, resulting in a fluorescence signal that remained constant over 24 h. These ThT-positive aggregates, however, have strikingly different morphologies, with the peptide 73–96 forming curvilinear structures, similar to protofibrils formed by the full-length JD (supplemental Fig. 2). In the presence of the peptide 73–96, fibril formation of both atx-3(14Q) and atx-3(78Q) was accelerated (*i.e.* lag times decreased, (Figs. 6*A* and 6*B*)) while the resulting fibril morphology appeared unchanged (Figs. 6*C*-6*F*). The peptide 73–96 exhibited a greater effect on the aggregation rate of atx-3(78Q) than for atx-3(14Q), as may be expected if this aggregation-prone region is more freely available in the former construct. No effect on atx-3 fibrillation was observed in the presence of the other JD peptides.

Together, the results presented suggest that atx-3(78Q) aggregates more rapidly than atx-3(14Q) because it exhibits altered conformational dynamics that enhance the exposure of the aggregation-prone segment (residues 73–96) of the JD and increase the probability of forming intermolecular interactions that promote amyloid assembly.

##### Oligomeric Species Undergo a Conformation Change during the Lag Phase of Assembly

**Fig. 7.**
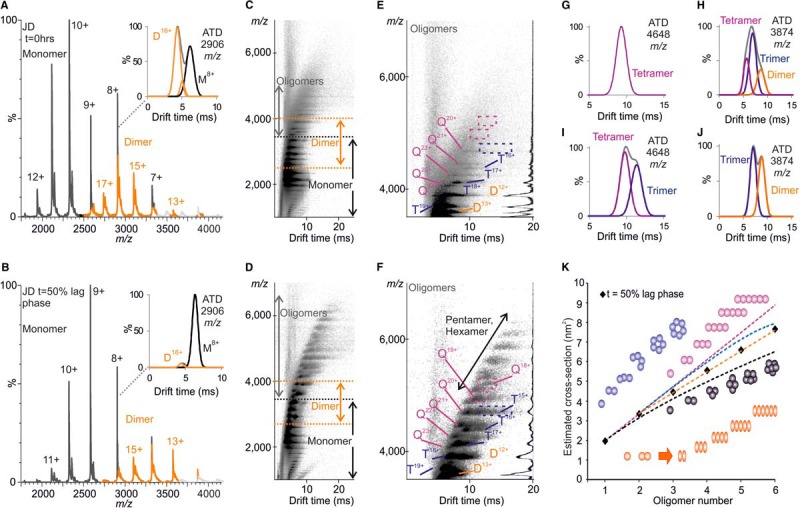
**JD oligomerization.** ESI-IMS-MS data obtained upon analysis of the JD (*top*) before aggregation (t = 0 h) and (*bottom*) at 50% through the lag phase of aggregation (∼8 h), as monitored by ThT fluorescence. (*A, B*) Representative ESI mass spectra with insets showing extracted ATDs for the *m/z* 2906 ion species. Monomer (black), D = dimer (orange). (*C, D*) Driftscope plots of (*A*) and (*B*), respectively. (*E, F*) Driftscope plots showing the oligomeric species in (*C*) and (*D*), respectively with MS spectra inset, T = trimer (blue) and Q = tetramer (purple). Dashed boxes highlight trimeric and tetrameric species (*E, inset*) absent at 0 h incubation and (*F, inset*) present at 50% through the lag phase of aggregation. Extracted ATDs for (*G, I*) the *m/z* 4648 ion species and (*H, J*) the *m/z* 3874 ion species at 0 h and 50% of the lag phase, respectively. (*K*) Oligomers increase in CCS with oligomer number in a linear fashion. Experimentally derived CCSs (black diamonds). Various models of oligomer assembly are shown: ring (blue), linear (purple), isotropic (black), and linear after dimerization (orange). The data best fit a model of linear assembly after dimerization (orange dashed line).

The population and conformational characteristics of oligomers present during the lag phase of amyloid assembly for the JD, atx-3(14Q) and atx-3(78Q) were next monitored using ESI-IMS-MS. This analysis revealed that a shift in the CSDs observed for monomeric and dimeric species of the JD occurred over time (Figs. 7*A* and 7*B*); more extended monomeric species (at higher charge states, 10+, 11+ and 12+) were depleted relative to monomeric species at lower charge states (7+, 8+, 9+) between 0 h and the midpoint of the lag phase and compact dimeric species (at lower charge states) increased in population (relative to dimeric species at higher charge states). At 0 h incubation, the *m/z* 2906 ion species of the JD consisted of a mixture of dimeric (16+) and monomeric (8+) species, but by 50% through the lag phase, this dimeric ion species was barely detectable (Figs. 7*A* and 7*B*, *inset*). This was accompanied by the appearance of pentamer and hexamer and an increased population of dimer to tetramer (Figs. 7*C–7F*).

The charge state distributions of trimeric and tetrameric species of the JD were also observed to change between 0 h and the midpoint of the lag phase. Lower-charged trimeric species (*e.g. m/z* 4648 trimer 15+) and tetrameric species (*e.g.* tetramer 19+ and 18+) became visible over time (Figs. 7*E–7I*) concomitant with higher-charged species being depleted (*e.g. m/z* 3874 tetramer 24+) (Figs. 7*H* and 7*J*). The largest population of oligomers (monomer to hexamer) was detected between 7 and 9 h of incubation (50% lag phase). By the end of the lag phase, higher-order oligomers were no longer detectable and only free monomer and dimer could be seen (supplemental Fig. 3).

In agreement with observations made for the JD, a shift in the monomeric, dimeric, and trimeric CSDs of atx-3(14Q) and atx-3(78Q) was observed to occur over the incubation time with more extended monomeric conformations becoming depleted as more compact oligomeric species formed (supplemental Fig. 4). Oligomers were also observed to form more rapidly for atx-3(78Q) than atx-3(14Q), consistent with the decreased lag time of atx-3(78Q) aggregation. Again, the largest population of oligomeric species (monomer to trimer) was observed at the midpoint of the lag phase of assembly for each construct.

These observations suggest that oligomers of the JD, atx-3(14Q), and atx-3(78Q) are formed via equivalent processes, with more compact conformations of dimeric and higher-order oligomeric species becoming relatively more highly populated during the lag phase of aggregation. As only monomers, dimers, and trimers are observed for atx-3(14Q) and atx-3(78Q), there are insufficient data to fit a model directly for these proteins. The same changes in the charge state distributions of the three species through the lag phase of aggregation are observed, however, and the protofibril core is the same for each species, suggesting a similar process is being undertaken. If so, then this could reflect conformational changes in the JD, previously observed by others to occur during oligomerization and suggested to be the first step on the aggregation pathway (19). To explore this in more detail, CCSs were estimated from the IMS-MS data for the most compact form of each JD oligomer observed after 50% of the lag phase of aggregation. The oligomers were observed to increase in CCS with increasing oligomer number in a linear fashion (Fig. 7*K*). The estimated CCSs were compared with those expected based on different models of assembly: isotropic, linear, ring, and linear subsequent to dimerization (see Methods). The data fit best to a model in which a linear increase in CCS with oligomer number is observed only after dimerization. This model suggests that there is a conformational change upon dimer formation, or within the dimer, that results in a template for further monomer addition.

## DISCUSSION

**Fig. 8.**
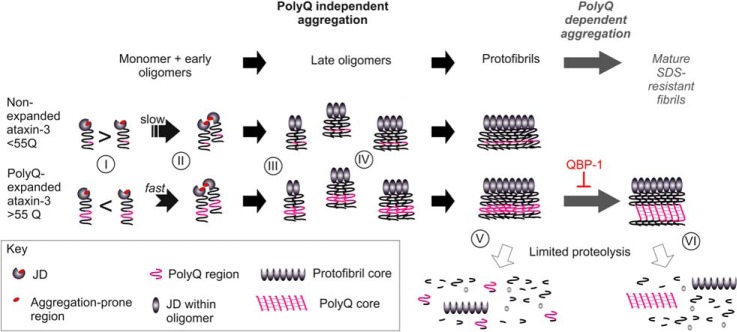
**Model of the mechanism of fibril formation for non-expanded and polyQ-expanded atx-3.** Monomeric atx-3 exposes its aggregation-prone region (residues 73–96), situated within the JD, transiently during native protein dynamics. In polyQ-expanded atx-3 these dynamic motions are perturbed, exposing this aggregation-prone region for longer time periods (I) and resulting in faster oligomerization (II). A conformational change within the JD occurs upon dimer formation, or within the dimer, that provides a template for further monomer addition (III). Oligomers increase in CCS with subunit number in a linear fashion after dimerization (IV). Protofibrils are formed in which the core involves JD residues between 62 and 182 (V). In polyQ-expanded atx-3, these protofibrils are able to mature into SDS-resistant fibrils by the formation of a network of intermolecular interactions between polyQ regions (**VI**). The two fibril cores are independent and structural rearrangement of the protofibril core is not required for mature fibril formation.

The results presented provide an increased understanding of atx-3 fibril formation *in vitro* and provide a rationale for the different rates of fibrillation observed for nonexpanded and polyQ-expanded atx-3. From these data, a detailed model for the mechanism of fibril formation can be proposed (Fig. 8).

Aggregation of atx-3 occurs initially through self-association of the JD from a native or near-native conformation (40, 41). Atx-3 with an expanded polyQ tract aggregates at a faster rate than nonexpanded atx-3, yet the presence of an expanded polyQ tract does not appear to affect the global stability or unfolding/folding rates of the JD (22). Results of the limited proteolysis experiments presented here suggest that the rate of conformational transitions between native-like states in the JD of atx-3(78Q) are altered such that residue K85 is more exposed for proteolysis (Fig. 8(I)). This results likely in exposure of the aggregation-prone region 73–96 for longer periods of time, increasing the probability of aberrant intermolecular interactions and thus enhancing the rate of aggregation (Fig. 8(II)). The peptide 73–96 forms protofibrils of similar morphology to those formed by atx-3, and this peptide also enhances the rate of atx-3 aggregation, suggesting that this region is involved in initial aggregate formation. Importantly, other peptides, although assembling into amyloid-like fibrils but with a different morphology, did not result in a rate enhancement. Previous studies have also shown that mutations within the 73–96 region of the JD (I77K/Q78K and W87K designed to reduce surface hydrophobicity (39) and S81A designed to increase stability (42)) result in reduced aggregation rates. How the presence of an expanded polyQ tract alters the conformational dynamics of the JD is unclear. The experiments presented here, and previous NMR experiments (43), provide no evidence for a stable interaction between the JD and the polyQ region. The polyQ region may therefore create a local denaturing environment, lowering the energy barrier for formation of an aggregation-prone native-like state and/or holding the JD in an aggregation-prone conformation for longer periods of time. This is not unprecedented as polyglutamine has been shown to have a urea-like affinity for unfolded polypeptides (44) and to reduce the global stability of neighboring protein domains in fusion constructs (45).

ESI-IMS-MS analysis of oligomers formed during the lag phase of atx-3 aggregation showed that a conformational change in the oligomer population occurred over time, such that more compact conformations became relatively more populated. Modeling of the CCSs of the JD oligomers observed suggests that a conformational change occurs at the dimer level (Fig. 8(III)). This is consistent with previous studies that showed that atx-3 aggregation can be seeded by the addition of preformed dimers and that these dimers display an increased β-sheet content and are recognized by the anti-oligomer antibody A11 (19). Here, using ESI-IMS-MS, we show that oligomerization of the JD was observed to proceed by a linear growth model after initial dimerization, consistent with the formation of elongated oligomeric assemblies (Fig. 8(IV)). Finally, analysis of the cores of protofibrils and mature SDS-resistant fibrils of atx-3 by use of limited proteolysis and MS analyses confirmed that the polyQ region is not structurally involved in protofibril formation (Fig. 8(V)). Formation of mature fibrils involves the independent polymerization of expanded polyQ regions, and our results show that this proceeds without requiring substantial alteration of the protofibril core (Fig. 8(VI)).

There is debate within the literature as to the mechanism of toxicity for all amyloid diseases, including Machado-Joseph disease. C-terminal fragments containing the polyQ tract, as well as the full-length protein, are found in neuronal inclusions (46), yet inclusion load does not correlate well with disease burden. FTIR spectroscopy experiments have provided evidence that the aggregation of polyQ-expanded atx-3 *in vivo* proceeds through the two-step mechanism observed *in vitro* (47), and soluble aggregates formed in the first stage of this process have been shown to be cytotoxic (47). The sites of ubiquitin binding (I77/Q78 and W87) (48) are contained within the aggregation-prone region of the JD (residues 73–96), suggesting that native functional interactions will ordinarily protect against aggregation (39). In the presence of an expanded polyQ tract, the enhanced propensity to aggregate may overcome these protective functional interactions. Indeed, nonexpanded atx-3 has been shown to form intranuclear inclusions under cell stress (49, 50). This highlights the importance of defining the nature of atx-3 conformational changes and oligomerization at the nanoscale level to understanding of the disease process. Here, we show how ESI-IMS-MS analyses can contribute toward a detailed understanding of aggregation processes, from monomer through to fibril, revealing unprecedented details of the mechanism of aggregation in atx-3 and rationalizing why an expanded polyQ tract enhances the rate of aggregation.

## Supplementary Material

Supplemental Data

## Footnotes

^1^ The abbreviations used are:

polyQ: polyglutamine
atx-3: ataxin-3
JD: Josephin domain
UIM: ubiquitin-interacting motif
atx-3(78Q): atx-3 with a pathological length polyQ tract of 77 glutamines (with a single, naturally occurring, lysine residue in the fourth position)
IMS: ion mobility spectrometry
TEM: transmission electron microscopy
CSD: charge state distribution
CCS: rotationally averaged collision cross-section
ATD: arrival time distribution
NMR: nuclear magnetic resonance
w/v: weight per volume
v/v: volume per volume
w/w: weight per weight
Ω: cross section
ThT: thioflavin T.
