# Supplementary material for: Examination of Ataxin-3 (atx-3) Aggregation by Structural Mass Spectrometry Techniques: A Rationale for Expedited Aggregation upon Polyglutamine (polyQ) Expansion
Source: Mol Cell Proteomics. 2015 Feb 20;14(5):1241–53. doi: 10.1074/mcp.M114.044610 (PMC4424396; doi:10.1074/mcp.M114.044610)
Supplement: Supplemental Data [file supp_14_5_1241__index.html]

Examination of ataxin-3 aggregation by structural mass spectrometry techniques: A rationale for expedited aggregation upon polyglutamine expansion — Examination of Ataxin-3 (atx-3) Aggregation by Structural Mass Spectrometry Techniques: A Rationale for Expedited Aggregation upon Polyglutamine (polyQ) Expansion — Effect of Polyglutamine Expansion on Ataxin-3 Aggregation — Supplemental Data 

# Examination of Ataxin-3 (atx-3) Aggregation by Structural Mass Spectrometry Techniques: A Rationale for Expedited Aggregation upon Polyglutamine (polyQ) Expansion

## Supplemental Data

**Files in this Data Supplement:**

- Supplementary Information - Supplementary Information to appear on-line
