# Supplementary material for: Examination of Ataxin-3 (atx-3) Aggregation by Structural Mass Spectrometry Techniques: A Rationale for Expedited Aggregation upon Polyglutamine (polyQ) Expansion
Source: Mol Cell Proteomics. 2015 Feb 20;14(5):1241–53. doi: 10.1074/mcp.M114.044610 (PMC4424396; doi:10.1074/mcp.M114.044610)
Supplement: Supplemental Data [file supp_M114.044610_mcp.M114.044610-1.docx]

**Examination of ataxin-3 aggregation by structural mass spectrometry techniques: A rationale for expedited aggregation upon polyglutamine expansion**

**Charlotte A. Scarff, Bruno Almeida, Joana Fraga, Sandra Macedo-Ribeiro, Sheena E. Radford, Alison E. Ashcroft**

**Supplementary Results**

**
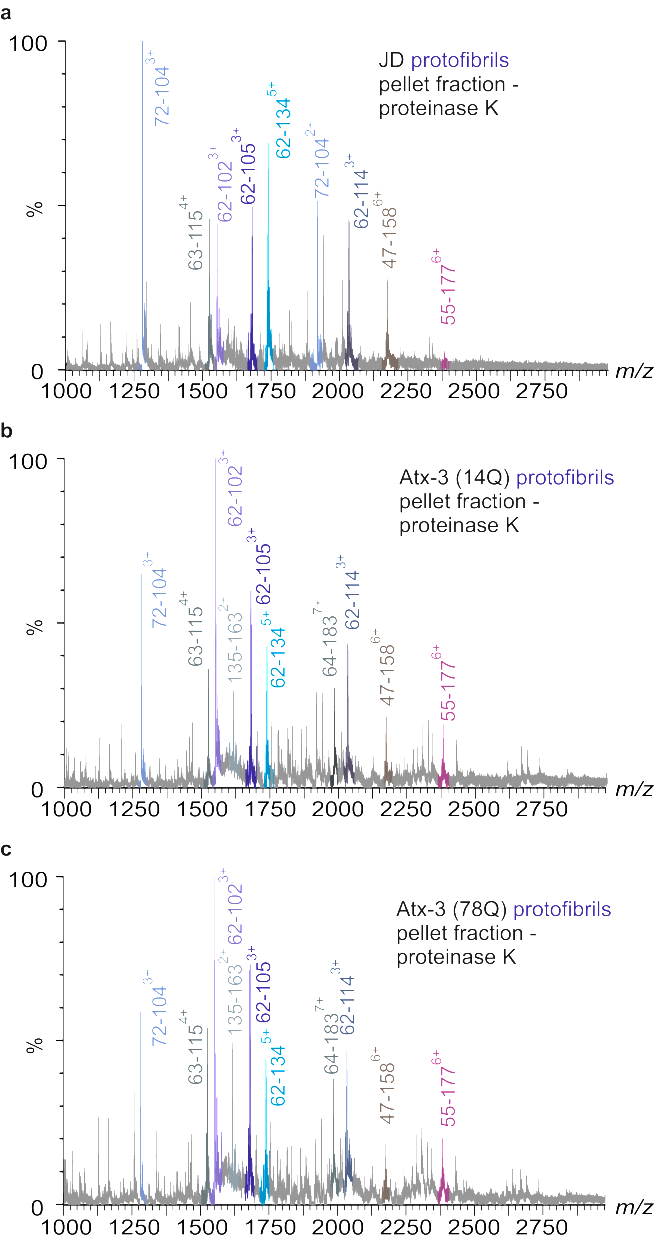
**

**Supplementary Figure 1. Limited proteolysis of protofibrils.** Mass spectra obtained of depolymerised pellet fractions obtained following limited proteolysis of (**a**) JD, (**b**) atx-3(14Q) and (**c**) atx-3(78Q) protofibrils with proteinase K. Colours are used to highlight the different peptide fragments identified.

**
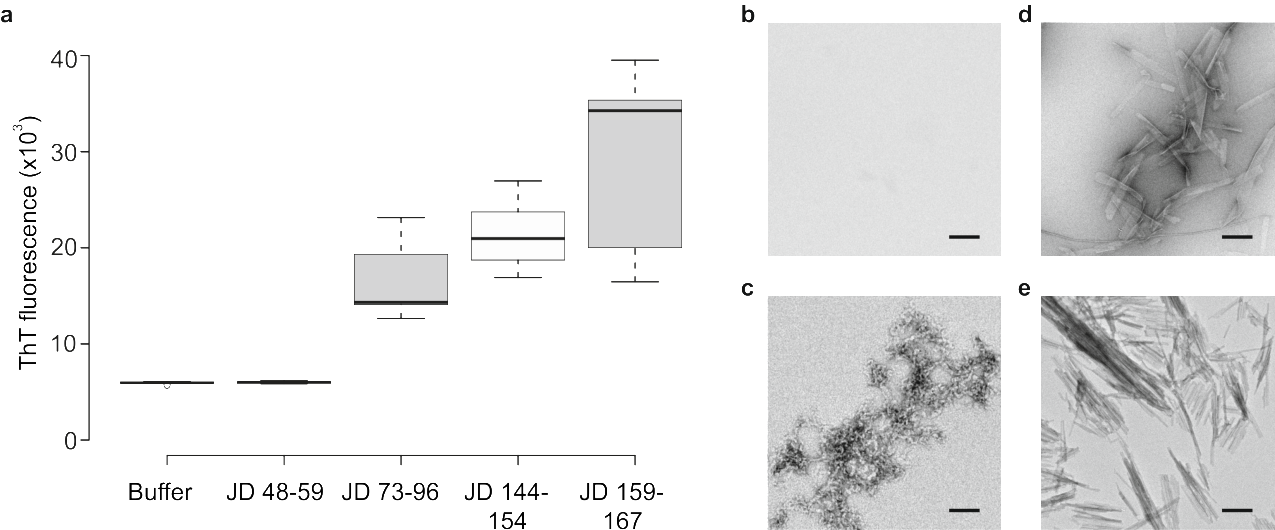
**

**Supplementary Figure 2. Aggregation behaviour of JD peptides.** (**a**) ThT fluorescence observed for JD peptides after quiescent incubation at 20 µM in 250 mM ammonium bicarbonate, 1 mM DTT, 20µM ThT, pH 8.1 for 24 hrs at 37 °C (n=5) and corresponding EM images for peptides (**b**) 48-59, (**c**) 73-96, (**d**) 144-154 and (**e**) 159-167. Scale bars = 500 nm. The peptide 73-96 formed curvilinear structures, similar to protofibrils formed by the full-length JD whereas the peptide 144-154 formed ribbon-like tubular structures and the peptide 159-167 formed rod-like structures. In comparison, a peptide consisting of JD residues 48-59, a region thought not to be involved in aggregation, did not form aggregates.

**
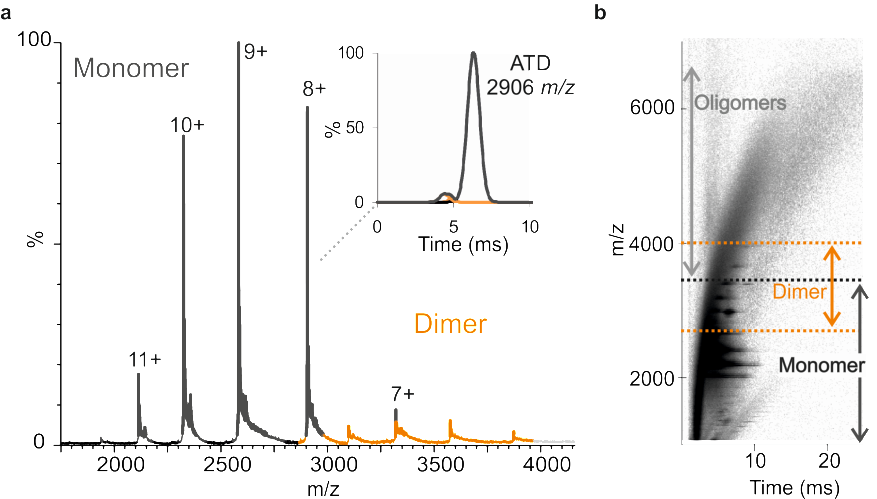
**

**Supplementary Figure 3. JD spectra at 100 % through the lag phase of aggregation.** (**a**) Mass spectrum obtained of the JD at 100 % through the lag phase of aggregation, (**a, inset**) extracted ATD for the m/z 2906 ion species and (**b**) the corresponding driftscope plot.


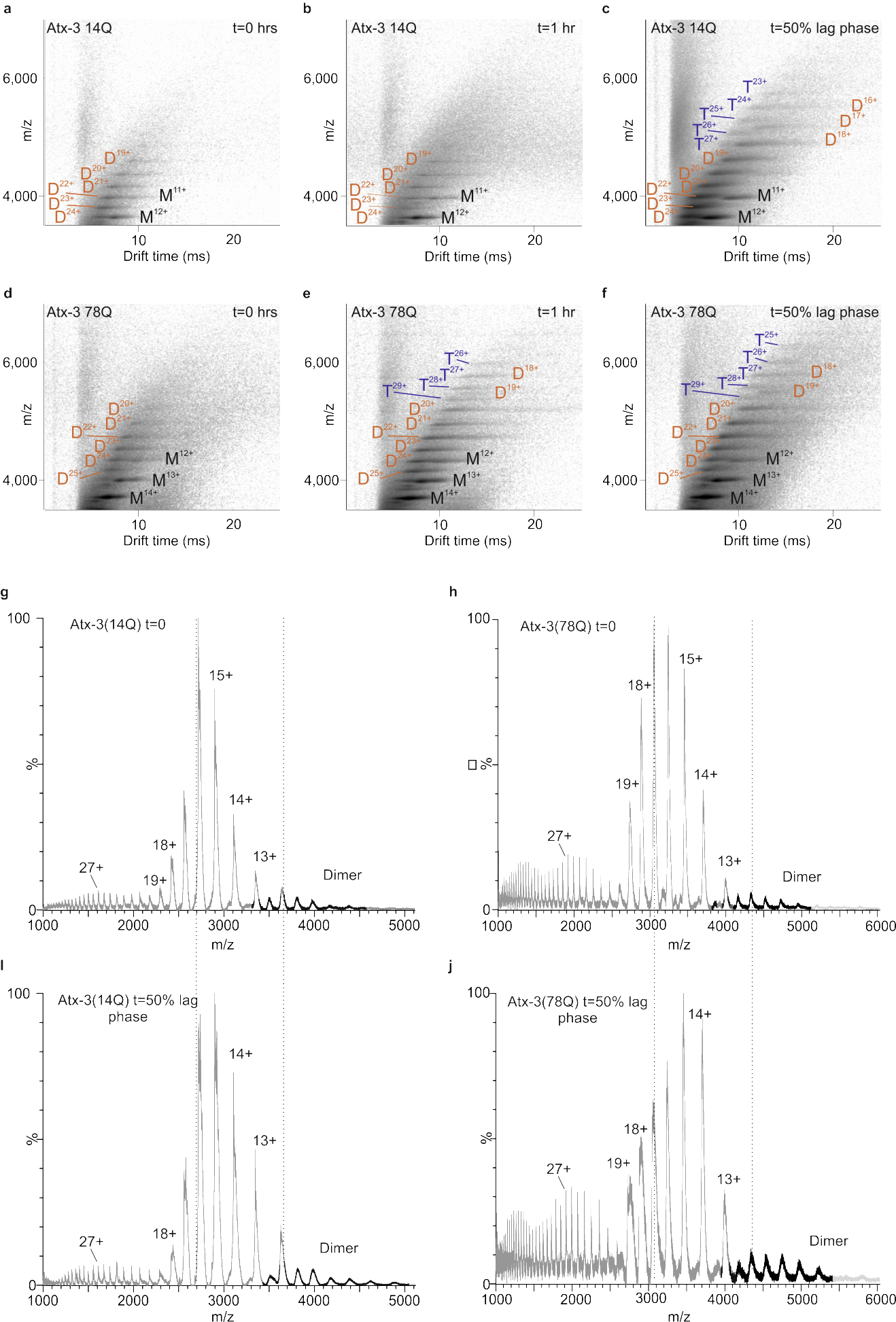


**Supplementary Figure 4. Oligomeric species of atx-3(14Q) and atx-3(78Q).** Driftscope plots obtained upon ESI-IMS-MS analysis of oligomeric species of atx-3(14Q) and atx-3(78Q) present at (**a, d**) 0 hr, (**b, e**) 1 hr and (**c, f**) 50% through the lag phase of each species (approximately 8 and 2 hrs), respectively. M = monomer, D = dimer, T = trimer and superscript = charge. Mass spectra obtained of (**g**) atx-3(14Q) and (**h**) atx-3(78Q) at 0 hr and (**i,j**) 50% through the lag phase of aggregation, respectively. A shift in the monomeric (grey) and dimeric (black) charge state distributions versus assembly time is observed.

**Supplementary Table 1. Products of limited proteolysis.** Summary of products of limited proteolysis identified for atx-3(14Q) and atx-3(78Q) after 15 minutes incubation at 37 °C with bovine trypsin (100:1 protein: trypsin molar ratio).
